# Sociodemographic, clinical and laboratory characteristics and risk factors for mortality of hospitalized COVID-19 patients at alternate care site: a Latin American experience

**DOI:** 10.1080/07853890.2023.2224049

**Published:** 2023-06-15

**Authors:** Jorge A. Alegría-Baños, Montserrat A. Rosas-Alvarado, José C. Jiménez-López, Marcos Juárez-Muciño, Carlos A. Méndez-Celis, Sharon T. Enríquez-De Los Santos, Rafael R. Valdez-Vázquez, Diddier Prada-Ortega

**Affiliations:** a Oncology Center, Medica Sur, Mexico City, Mexico; b General Directorate for the Provision of Medical Services and Emergencies, Mexico City Health Secretariat, Mexico City, Mexico; c Postgraduate in Earth Sciences, Institute of Geology, National Autonomous University of Mexico, Mexico City, Mexico; d Laboratory of Immunotherapy and Tissue Engineering, Faculty of Medicine, National Autonomous University of Mexico, Mexico City, Mexico; e Department of Medical Services, General Hospital 47 “Vicente Guerrero”, Mexican Social Security Institute, Mexico City, Mexico; f Medical Coordination, Temporary COVID-19 Unit “Centro Citibanamex”, Mexico City, Mexico; g Dirección de Investigación, Instituto Nacional de Cancerología, Mexico City, Mexico; h Department of Environmental Health Science, Columbia University Mailman School of Public Health, New York City, NY, USA; i Institute for Health Equity Research, Mount Sinai Hospital, New York City, NY, USA

**Keywords:** COVID-19, mortality, risk factors, alternate care sites, Latin America

## Abstract

**Background:**

The establishment of Alternate Care Sites (ACS) helped the most severely impacted countries expand their response capability. The aim of this study was to evaluate the clinical characteristics and risk factors associated with the mortality of hospitalized COVID-19 patients at Alternate Care Site in Mexico City.

**Patients and methods:**

A monocentric cohort study was conducted at Mexico City’s Temporary Unit COVID-19 (UTC-19). Sociodemographic, clinical, laboratory and treatment variables were included in the analysis.

**Results:**

A total of 4865 patients were included, with a mean age of 49.33 years ± SD 15.28 years (IQR 38 to 60 years); 50.53% were women. 63.53% of the patients presented at least one comorbidity, the most frequent being: obesity (39.94%), systemic arterial hypertension (25.14%), and diabetes mellitus (21.52%). A total of 4549 patients (93.50%) were discharged due to improvement, 64 patients (1.31%) requested voluntary discharge, 39 patients (0.80%) were referred to another unit, and 213 patients (4.37%) died. Factors that were independently and significantly associated with death included male gender (odds ratio [OR], 1.60), age ≥ 50 years (OR 14.75), null or low schooling (OR 3.47), have at least one comorbidity (OR 3.26), atrial fibrillation (OR 22.14). In the multivariate analysis, the lymphopenia ≤ 1 × 10^3/^μL (OR 1.91), and having required steroid treatment (OR 2.85), supplemental oxygen with high-flow nasal cannula (OR 3.12) or invasive mechanical ventilation (OR 42.52), was significantly associated with an increased risk of death.

**Conclusions:**

This study identified the clinical characteristics and risk factors for mortality of hospitalized COVID-19 patients at ACS in Mexico City.

**KEY MESSAGES**
- An Alternate Care Site (ACS) is any building or structure that is temporarily converted or constructed for healthcare use during a public health emergency.
- Factors associated with death included male gender, age over 50 years, and lower educational attainment (elementary school or less).
- The findings corroborate the utility of the CALL score as a predictor of mortality; lymphopenia ≤1 × 10^3^/μL was the most relevant biomarker.

## Introduction

Coronavirus disease 2019 (COVID-19) was reported in China and quickly spread throughout the world. A pandemic status was declared by the World Health Organization in March 2020. COVID-19 has caused over 390 million cases and over 5.7 million deaths [1].

As a result of the crisis, it was necessary to increase the medical response capacity by adapting hospital facilities and establishing temporary field hospitals in public places such as concert halls, hotels, sports stadiums, and convention centres [2–4]. More countries needed to establish Alternate Care Sites (ACS), which are buildings or structures that are temporarily transformed for sanitary use, as defined by the US Centers for Disease Control and Prevention [5]. ACS models for COVID-19 vary in terms of the type of care they provide, from those that care for patients with mild symptoms to those that provide acute care for patients who require ventilatory assistance [6–8].

The ACS allow for a quick response to hospital saturation while saving time and money; however, they pose a logistical and technological challenge and must adhere to quality standards [9]. Evidence of this is the patients’ concern and external marginalization because of a lack of medical personnel, inadequate equipment, and limited medical services.

In February 2020, China put the first three temporary hospitals (Fangcang, Huoshenshan, and Leishenshan) into operation in response to insufficient medical resources, limiting the national spread and reducing the number of deaths [10–13]. In the following weeks, various countries implemented similar measures, mainly in cities with the highest number of cases such as Madrid and Asturias in Spain [14,15], Warsaw in Poland [16], Cape Town in South Africa [17], Sao Paulo in Brazil [18] and New York in the United States of America [19].

Until December 2022, Mexico was the fifth country with the highest SARS-CoV-2 deaths, accounting for more than 333,000 deaths. Until October 2021, Mexico registered 283,122 accumulated cases of SARS-CoV-2 infection in health workers, with 4517 deaths [20,21].

The Temporary Unit COVID-19 (UTC-19) in Mexico City was one of the responses implemented to the crisis. It was installed in less than three weeks, with a surface area of 35,000 m^2^, to provide care to patients referred from the hospitals in the health system, to depressurize the demand for specialized care, and to maintain the hospital capacity in Mexico City. The UTC-19 began operations on 29 April 2020, with 238 beds and eight critical care units dedicated to convalescent and recovery patients. However, in response to increased demand, on 1 June 2020, it began an early hospitalization program for patients with COVID-19 who also have risk factors for progression to severity, such as obesity, diabetes mellitus, or arterial hypertension, and who were referred by strategic primary evaluation centres.

UTC-19 will release 112,555 days/bed from the Mexico City hospital network until its closure on 15 June 2021, with an expansion to 455 general care beds, 80 beds with high-flow oxygen, 54 intensive care beds, and 18 beds for post-critical care. More than 3481 health professionals took part in the study, which included 428,384 laboratory and X-ray tests, 52,988 patient follow-up calls, and 9698 staff training sessions [22].

The aim of this study was to evaluate the clinical characteristics and risk factors associated with the mortality of hospitalized COVID-19 patients at Alternate Care Site in Mexico City. We explored the clinical, sociodemographic, biochemical, and therapeutic characteristics with predictive value for mortality, through two multivariate models.

## Methodology

### Study design and population

A cohort study in a single centre was performed in the Temporary Unit COVID-19 (UTC-19) of the Citibanamex Center in Mexico City. Inclusion criteria were patients ≥ 16 years old, hospitalized in the UTC-19 from 29 April 2020 to 18 May 2021, who were admitted with COVID-19 confirmed microbiologically by reverse transcription-polymerase chain reaction (RT-PCR) through a nasopharyngeal sample [23,24]. Exclusion criteria were denial or withdrawal of informed consent. Patients were treated at their physician’s clinical judgement, according to local protocols based on international recommendations.

### Outcomes

The primary outcome was the mortality rate during hospitalization. The secondary outcome was the sociodemographic, clinical, and laboratory characteristics and risk factors for mortality. We established a risk model for patient mortality through risk factor analysis. The follow-up period was established from admission to discharge (due to improvement, voluntary discharge, or reference to another unit), or death.

### Data collection

Data was collected using the Research Electronic Data Capture (REDCap) platform, an open architecture web application, for building and managing online surveys and databases, specifically geared for research studies and operations, which requires a licence to gain access to the source code. Data collected through REDCap was verified by UTC-19 staff periodically.

Sociodemographic characteristics included sex, age (aging groups using >50, >60, and >70 years old as cutpoints), and education level. The clinical symptoms included fever, cough, sore throat, rhinorrhoea, myalgia, headache, arthralgia, altered consciousness/confusion, nausea/vomiting, and diarrhoea. Clinical characteristics were the body mass index (BMI), comorbidities (diabetes, arterial hypertension, coronary disease, atrial fibrillation, and alcohol consumption), and their respective counting data. Biochemical and physiological characteristics included D-dimer levels, gasometric values, blood cell count, and their ratios.

We used NEWS (National Early Warning Score) and CALL (C: comorbidity, A: age, L: lymphocyte level, L: lactate dehydrogenase level, LDH), which are validated scores to predict the disease progression or mortality due to COVID-19 [25,26]. The numerical and operational values of these scores were used. For the NEWS score, ≤ 4 points predicted a low clinical risk, 5–6 points a medium clinical risk, and ≥ 7 points a high clinical risk. For the CALL score, 4–6 points indicated a low progression risk, and ≥ 7 points predicted a high progression risk.

We include high-flow nasal cannula (HFNS) and invasive mechanical ventilation (IMV) in the treatment variables. The statistically significant variables for association with the outcome variable (deceased patients) were included in the uni- and multivariate binomial logistic regression analyses.

The study was performed following the guidelines of the General Health Law on Research for Health - Mexico, the Declaration of Helsinki, and the ICH-Good Clinical Practices. The study was approved by the Research Ethics Committee (FM/DI/098/2020), of the Research Division of the National Autonomous University of Mexico (Supplementary Material, Figure S1). Informed consent was obtained from all patients.

### Statistical analysis

Continuous measurements are presented as the mean and standard deviation (SD) or as the median and interquartile range (IQR), and categorical variables are expressed as a percentage of the total number of observations. For laboratory results, it was evaluated whether the measurements were outside the normal range. Chi-square tests were used for counts, analysis of variance, Welch’s T test for numerical parameters, and the Wilcoxon test for position values. The odds ratios were reviewed using univariate and multivariate models with the variables in which important differences were found in terms of the proportion per group of death. Statistical analysis was performed using R software (version 3.6.3).

## Results

### Sociodemographic characteristics

A total of 4865 patients admitted between 29 April 2020 and 18 May 2021, diagnosed with COVID by a confirmatory test with real-time RT-PCR, were included. 51.53% were women. The mean age was 49.33 years ± SD 15.28 (IQR 38 to 60 years). A total of 4549 patients (93.50%) were discharged due to improvement, 64 patients (1.31%) requested voluntary discharge, 39 patients (0.80%) were referred to another unit, and 213 patients (4.37%) died. The 95% confidence interval for mortality using a binomial distribution was 3.82–4.99%. The mortality showed an important variation along the months with a maximum value of 7.14% during January 2021.

Table 1 shows the sociodemographic characteristics and medical history of the patients. The group of patients who died with those who survived the infection was compared. Significant differences were found for the fatal outcome by gender (*p* = .0011) and age (*p* < .0001). The most common schooling degrees were undergraduate (32.23%) and baccalaureate (27.68%). The patients who had zero (0.88%) or elementary (12.74%) education presented higher mortality percentages within their groups, with a significant difference with respect to the other grades (*p* = .006). 63.53% of the patients presented at least one comorbidity, the most frequent being: obesity (39.94%), systemic arterial hypertension (25.14%), and diabetes mellitus (21.52%). The body mass index in both trend and position values or by intervals did not show a significant difference between the groups when comparing overweight or obesity versus normal weight or underweight (*p* = .5677).

**Table 1.** Baseline demographic characteristics of hospitalized COVID-19 patients who required admission to the UTC-19, México City (*n* = 4864).

Variable	Levels or measurements	Total n (%)	Survivor n (%)	Non-survivor n (%)	*p*
Sex	Woman	2507 (51.531)	2421 (96.569)	86 (3.430)	**.0011**
	Man	2358 (48.468)	2231 (94.614)	127 (5.385)	
Age (years)	Mean (SD)	49.330 (15.289)	48.566 (15.006)	65.9577 (11.478)	**<.0001**
	Median (IQR)	50 (38–60)	50 (37–59)	66 (57–73)	**<.0001**
	>50	2395 (49.229)	2197 (91.732)	198 (8.267)	**<.0001**
	>60	1130 (23.227)	985 (87.168)	145 (12.831)	**<.0001**
	> 0	386 (7.934)	316 (81.865)	70 (18.134)	**<.0001**
School degree	Bachelor’s degree	1568 (32.230)	1500 (95.663)	68 (4.336)	**.0006**
	High school	1347 (27.687)	1300 (96.510)	47 (3.489)	
	Middle school	912 (18.766)	874 (95.728)	39 (4.271)	
	Elementary school	620 (12.744)	576 (92.903)	44 (7.096)	
	Post-graduate	168 (3.453)	160 (95.238)	8 (4.761)	
	Null	43 (0.883)	37 (86.046)	6 (13.953)	
Comorbidities	Nothing	1773 (36.464)	1741 (98.139)	33 (1.860)	**<**.**0001**
	One	1767 (36.320)	1686 (95.416)	81 (4.584)	
	Two	862 (17.718)	795 (92.227)	67 (7.772)	
	Three or above	392 (8.057)	364 (92.857)	28 (7.142)	
	Overweight or obesity (BMI ≥ 25 kg / m^2^)	3956 (81.315)	3780 (81.255)	176 (82.629)	.**5677**
	Obesity(BMI ≥ 30 kg / m^2^)	1943 (39.946)	1858 (39.399)	85 (39.906)	1
	Alcohol consumption	1725 (35.457)	1664 (35.769)	61 (28.638)	**.0393**
	Tobacco consumption	1430 (29.393)	1366 (29.363)	64 (30.046)	.8956
	Arterial hypertension	1223 (25.138)	1116 (23.989)	107 (50.234)	**<.0001**
	Diabetes mellitus	1047 (21.521)	961 (20.657)	86 (40.375)	**<.0001**
	Pneumopathy	148 (3.042)	139 (2.988)	9 (4.225)	.4098
	Asthma	110 (2.261)	107 (2.300)	3 (1.408)	.5351
	Depression or anxiety	105 (2.158)	103 (2.214)	2 (0.939)	.3119
	Autoimmune disease	70 (1.438)	66 (1.418)	4 (1.877)	.7979
	Coronary disease	51 (1.048)	44 (0.945)	7 (3.286)	**.0033**
	Hepatic disease	30 (0.616)	29 (96.666)	1 (3.333)	1
	Kidney disease - YES n (%)	20 (0.411)	17 (0.365)	3 (1.408)	.0753
	Active cancer	20 (0.411)	17 (0.365)	3 (1.408)	.0753
	HIV infection	19 (0.390)	19 (0.408)	0 (0)	.7093
	Atrial fibrillation	6 (0.123)	3 (0.065)	3 (1.408)	**<.0001**
IMC	Mean (SD)	28.793 (4.760)	28.788 (4.774)	28.894 (4.429)	.7349

*Note:* BMI: body mass index; IQR: interquartile range; SD: standard deviation.

### Clinical features

Table 2 shows the clinical characteristics and grouping based on the NEWS and CALL scales. The most frequent clinical manifestations were headache (62.87%), myalgia (62.69%), cough (61.97%), arthralgia (53.03%), and fever (49.94%). The mean time elapsed between the onset of symptoms and admission was 6.9 days. Most of the patients attended (81.27%) had low clinical risk (NEWS ≤ 4); 50.52% were classified as low risk of progression evaluated with CALL.

**Table 2.** Baseline clinical characteristics of hospitalized COVID-19 patients who required admission to the UTC-19, México City (*n* = 4864).

Variable	Levels or measurements	Total n (%)	Survivor n (%)	Non-survivor n (%)	*p*
Symptoms	Headache	3059 (62.877)	2942 (63.241)	117 (54.929)	**.0172**
	Myalgia	3050 (62.692)	2917 (62.704)	133 (62.441)	.9959
	Cough	3015 (61.973)	2864 (61.564)	151 (70.892)	**.0076**
	Arthralgias	2580 (53.031)	2449 (52.644)	131 (61.502)	**.0138**
	Fever	2388 (49.948)	2288 (49.183)	100 (46.948)	.5701
	Pharyngodynia	1727 (35.498)	1673 (35.963)	54 (25.352)	**.0020**
	Anosmia	1203 (24.732)	1163 (25.000)	40 (18.779)	**.0481**
	Rhinorrhoea	1114 (22.898)	1074 (23.086)	40 (18.779)	.1677
	Dysgeusia	1083 (22.261)	1045 (22.463)	38 (17.940)	.1331
	Diarrhoea	770 (15.827)	738 (15.864)	32 (15.023)	.8160
	Conjunctivitis	381 (7.831)	367 (7.889)	14 (6.572)	.5695
Days between onset of symptoms and admission	Mean (SD)	6.9517 (5.631)	7.0017 (5.686)	5.8592 (4.093)	**.0001**
	Median (IQR)	6 (4–9)	6 (4–9)	5 (3–7)	.**0003**
Progression risk	Low (CALL 4–6)	2458 (50.524)	2429 (98.820)	29 (1.179)	**<.0001**
	High (CALL ≥ 7)	2406 (49.475)	2223 (92.355)	184 (7.644)	
Clinical risk	Low (NEWS ≤ 4)	3954 (81.274)	3814 (96.459)	140 (3.504)	**<.0001**
	Average (NEWS 5–6)	671 (13.792)	621 (92.548)	50 (7.451)	
	High (NEWS ≥ 7)	240 (4.932)	217 (90.416)	23 (9.583)	
CALL	Mean (SD)	6.6212 (2.301)	6.4991 (2.230)	9.2864 (2.208)	**<.0001**
	Median (IQR)	6 (4–8)	6 (4–8)	9 (8–11)	**<.0001**
NEWS	Mean (SD)	3.2545 (1.812)	3.2081 (1.766)	4.2676 (2.418)	**<.0001**
	Median (IQR)	3 (2–4)	3 (2–4)	4 (3–5)	**<.0001**

CALL: Comorbidity − Age − Lymphocyte Count − Lactate dehydrogenase Score; IQR: interquartile range: NEWS: National Early Warning Score; SD: standard deviation.

Regarding the scores of the clinical risk and risk of progression scales, differences were also found in terms of groups by death, both in their scores and in the categories (CALL: *p* < .0001, 95% CI: 2.48–3.09 points less in average for the surviving group; NEWS: *p* < .0001; 95% CI: 0.72–1.39 points lower on average for the surviving group).

### Laboratory features

In relation to the laboratory data (Table 3), when analysing the biomarkers of inflammation, significant differences (*p* < .0001) were found for the group with fatal outcome, in the mean values of the serum concentrations of lactic dehydrogenase and D-dimer, reaching 1.2 times the level found in the group without death. There was no significant difference in the levels of biomarkers such as ferritin, C-reactive protein, and creatine phosphokinase (CPK) between the study groups.

**Table 3.** Comparison of laboratory data and treatment by outcome (*n* = 4865).

Characteristics	Total	Survivor	Non-survivor	*p*
Inflammatory markers				
CPK (U/L)^a^	37 (24–65)	36 (24–64)	39 (24–68.5000)	.2571
LDH (UI/L)^a^	193 (156–249)	192 (156–247)	223 (182–309)	**<.0001**
D-Dimer (ng/mL)^a^	390 (240–640)	380 (240–630)	460 (320–840)	**<**.**0001**
High-sensibility C protein (mg/L)^b^	46.443 (68.592)	46.517 (68.508)	45.158 (70.302)	.7839
Ferritin (ng/dL)^a^	329.2 (150.7–631.2)	330.3 (152.6–632.7)	309.4 (126.425–593.175)	.3975
Complete blood count				
Haemoglobin (g/dL)^b^	14.6234 (1.605)	14.622 (1.599)	14.656 (1.725)	.7754
White blood cell (1 × 10^3^/μL)^a^	5.6 (4.4–7.3)	5.6 (4.4–7.3)	6.3 (5–9.4)	**<.0001**
Lymphocytes (1 × 10^3^/μL)^a^	1.165 (0.836–1.584)	1.186 (0.855–1.606)	0.7748 (0.54–1.091)	**<.0001**
Neutrophils (1 × 10^3^/μL)^a^	3.715 (2.653–5.429)	3.674 (2.629–5.348)	5.015 (3.575–7.82)	**<.0001**
Monocytes (1 × 10^3^/μL)^a^	0.4235 (0.303–0.57)	0.4256 (0.307–0.57)	0.3850 (0.226–0.586)	**.0141**
Eosinophils (1 × 10^3^/μL)^b^	0.618 (1.257)	0.636 (1.272)	0.231 (0.748)	**<.0001**
Platelets count (1 × 10^3^/μL)^a^	211 (169–270)	213 (170–271)	180 (147–221)	**<.0001**
Serological biomarkers^a^				
Monocyte-lymphocyte ratio (MLR)	348.9736 (245.238–512.195)	345.408 (243.573– 504.201)	481.2968 (333.333–708.661)	**<.0001**
Neutrophile-lymphocyte ratio (NLR)	3.158 (1.943–5.556)	3.073 (1.902–5.297)	6.654 (3.865–12.743)	**<.0001**
Derivated neutrophile-lymphocyte ratio (dNLR)	0.885 (0.840–0.926)	0.883 (0.8384–0.923)	0.931 (0.881–0.957)	**<.0001**
Platelets-lymphocyte ratio	183.419 (129.776–266.335)	182.082 (128.885–261.920)	231.125 (156.045–363.488)	**<.0001**
Nutritional index	364.028 (303.198–414)	363.5 (303.276–413.442)	371.918 (302.812–421.474)	.5116
Blood gas analysis^b^				
O_2_ saturation (%)	94.1470 (3.797)	94.1575 (3.823)	93.9005 (3.205)	.2714
PaO_2_ (mmHg)	73.1120 (18.353)	73.1826 (18.299)	71.6550 (19.434)	.2752
PaCO_2_ (mmHg)	33.3364 (3.858)	33.3764 (3.848)	32.5119 (3.985)	**.0029**
PaO_2_/FiO_2_ (%)	298.1367 (0.903)	299.8460 (0.901)	262.8328 (0.882)	**<.0001**
Hepatic/renal function				
Albumin (g/dL)^b^	3.5048 (0.715)	3.5019 (0.714)	3.5481 (0.728)	.3739
Total bilirubin (mg/dL)^b^	0.591 (0.450)	0.592 (0.456)	0.571 (0.363)	.4461
Glucose (mg/dL)^b^	134.810 (53.090)	134.755 (53.154)	135.720 (52.108)	.7947
AST (U/L)^a^	26 (19–40)	26 (19–40)	27 (19–42.75)	.7294
ALT (U/L)^a^	33 (22–56)	33 (22–56)	32 (21–51)	.2715
Creatinine (mg/dL)^a^	0.879 (0.659)	0.882 (0.662)	0.821 (0.598)	.1528
Treatments^c^				
Steroid use	3117 (64.0699)	2929 (62.9622)	188 (88.2629)	**<.0001**
High flow nasal cannula	953 (19.588)	787 (16.917)	166 (77.934)	**<.0001**
Invasive mechanical ventilation	405 (8.324)	225 (4.836)	180 (84.507)	**<.0001**

^a^Median and interquartile range (IQR), tested by Wilconxon-Mann-Whitney U test.

^b^Mean and standard deviation, tested by Welch’s T test.

^c^Counts and percentage, tested by Chi-squared test with Yates’ continuity correction.

The values in boldface fonts are used for the correspondent significant (*p* < 0.05) probability tests.

Significant differences were found by outcome group in platelet counts (*p* < .0001), leukocytes (*p* < .0001), lymphocytes (*p* < .0001), neutrophils (*p* < .0001) and eosinophils (*p* < .0001). In turn, some of the indices studied as serum biomarkers also showed significant differences, especially the monocyte-lymphocyte and neutrophil-lymphocyte ratios, in which increases of between 1.5 and 3.8 times the values were found in the group with deaths with respect to what was found in the group without deaths (*p* < .0001). The nutritional index of the patients did not show a significant difference between the groups (*p* = .511). Finally, the blood gas analysis showed a significant difference in PaCO_2_ values (*p* = .0029) and in the PaO_2_/FiO_2_ ratio (*p* < .001) between both groups.

### Treatment

64.06% of the patients received steroids as part of the pharmacological treatment (Table 3); 19.58% required supplemental oxygen through high-flow nasal tips (HNFC) and 8.32% required invasive mechanical ventilation (IMV). Each of these three variables showed a statistically significant difference between the groups of survivors and deaths (*p* < .0001).

### Univariate and multivariate analysis

Table 4 shows the characteristics that were included in the univariate and multivariate analyses to identify factors associated with the death of the patients. Estimates were made using profile intervals. Performance data on optimal cut-off values for numerical variables of this study are presented in Supplementary Table S1.

**Table 4.** Odds ratio and 95% confidence intervals of baseline, clinical, biochemical, and treatment characteristics with predictive value for mortality (*n* = 4865).

Baseline characteristics		Survivor n (%)	Non-survivor n (%)	OR (univariate)	OR (multivariate)^a^
Sex	Male	2231 (48.0)	127 (59.6)	**1.60 (1.21**–**2.13, *p* =.001)**	**1.85 (1.36**–**2.53, *p* =.0001)**
	Female	2421 (52.0)	86 (40.4)		
Age	≥50 years	2197 (47.2)	198 (93.0)	**14.75 (9.01**–**26.11, *p* <.001)**	**9.36 (5.60**–**16.83, *p* <.0001)**
	<50 years	2455 (52.8)	15 (7.0)		
Comorbidities	One or more	2846 (62.0)	176 (84.2)	**3.26 (2.27**–**4.83, *p* <.001)**	1.59 (1.00–2.55, *p* = .050)
	Without	1741 (38.0)	33 (15.8)		
Alcohol consumption	Yes	1664 (35.8)	61 (28.6)	**0.72 (0.53**–**0.97, *p* = .034)**	0.76 (0.54–1.05, *p* = .100)
	No	2985 (64.2)	152 (71.4)		
Atrial fibrillation	Yes	3 (0.1)	3 (1.4)	**22.14 (4.08**–**120.23, *p* < .001)**	**7.15 (1.07**–**51.41, *p* = .039)**
	No	4649 (99.9)	210 (98.6)		
Coronary disease	Yes	44 (0.9)	7 (3.3)	**3.56 (1.45**–**7.51, *p* = .002)**	1.64 (0.65–3.59, *p* = .251)
	No	4608 (99.1)	206 (96.7)		
Arterial hypertension	Yes	1116 (24.0)	107 (50.2)	**3.20 (2.42**–**4.22, *p* < .001)**	1.07 (0.76–1.51, *p* = .715)
	No	3536 (76.0)	106 (49.8)		
Diabetes mellitus	Yes	961 (20.7)	86 (40.4)	**2.60 (1.96**–**3.44, *p* < .001)**	0.93 (0.67–1.30, *p =* .688)
	No	3691 (79.3)	127 (59.6)		
Arthralgias	Yes	2449 (52.6)	131 (61.5)	**1.44 (1.09**–**1.91, *p* = .012)**	**1.55 (1.15**–**2.09, *p* = .004)**
	No	2203 (47.4)	82 (38.5)		
Cough	Yes	2864 (61.6)	151 (70.9)	**1.52 (1.13**–**2.07, *p* = .006)**	**1.41 (1.04**–**1.95, *p* = .032)**
	No	1788 (38.4)	62 (29.1)		
Clinical risk	High (NEWS ≥ 7)	217 (4.7)	23 (10.8)	**2.89 (1.78**–**4.50, *p* < .001)**	**1.86 (1.07**–**3.07, *p* = .020)**
	Medium (NEWS 5–6)	621 (13.3)	50 (23.5)	**2.19 (1.56**–**3.04, *p* < .001)**	**1.68 (1.17**–**2.37, *p* = .004)**
	Low (NEWS ≤ 4)	3814 (82.0)	140 (65.7)		
Progression risk	High (CALL ≥ 7)	2223 (47.8)	184 (86.4)	**6.93 (4.75**–**10.50, *p* < .001)**	**2.57 (1.59**–**4.15, *p* < .001)**
	Low (CALL 4–6)	2429 (52.2)	29 (13.6)		
Laboratory findings and treatments		Survivor n (%)	Non-survivor n (%)	OR (univariable)	OR (multivariable)^b^
D-dimer (mg/mL)	> 500	1637 (35.3)	96 (45.1)	**1.51 (1.14**–**1.98, *p* = .004)**	1.12 (0.78–1.63, *p* = .533)
	≤ 500	3003 (64.7)	117 (54.9)		
PaO_2_ / FiO_2_(ratio × 100)	≤ 300	2053 (49.2)	137 (67.8)	**2.18 (1.62**–**2.96, *p* < .001)**	0.70 (0.46–1.05, *p* = .085)
	> 300	2119 (50.8)	65 (32.2)		
White blood cell (1 × 10^3^/μL)	> 10	403 (8.7)	46 (21.6)	**2.90 (2.04**–**4.05, *p* < .001)**	0.88 (0.52–1.47, *p* = .630)
	≤ 10	4241 (91.3)	167 (78.4)		
Lymphocytes (1 × 10^3^/μL)	≤ 1	1669 (35.9)	150 (70.4)	**4.24 (3.16–5.77, *p* < .001)**	**1.91 (1.15**–**3.18, *p* = .012)**
	> 1	2975 (64.1)	63 (29.6)		
MLR	> 200	3975 (85.6)	193 (91.0)	**1.70 (1.08**–**2.84, *p* = .029)**	0.80 (0.43–1.52, *p* = .475)
	≤ 200	666 (14.4)	19 (9.0)		
NLR	>5	1274 (27.4)	133 (62.4)	**4.40 (3.32**–**5.87, *p* < .001)**	1.60 (0.91–2.80, *p* = .100)
	≤ 5	3370 (72.6)	80 (37.6)		
dNLR	> 0.88	2437 (52.5)	160 (75.1)	**2.73 (2.01**–**3.78, *p* < .001)**	0.84 (0.52–1.37, *p* = .495)
	≤ 0.88	2207 (47.5)	53 (24.9)		
PLR	>200	1978 (42.6)	124 (58.2)	**1.88 (1.42**–**2.49, *p* < .001)**	0.62 (0.37–1.04, *p* = .072)
	≤ 200	2666 (57.4)	89 (41.8)		
Steroid use	Yes	2929 (63.0)	188 (88.3)	**4.42 (2.96**–**6.90, *p* < .001)**	**2.85 (1.57**–**5.47, *p* = .001)**
	No	1723 (37.0)	25 (11.7)		
High flow nasal cannula	Yes	787 (16.9)	166 (77.9)	**17.35 (12.54**–**24.44, *p* < .001)**	**3.12 (1.99**–**4.97, *p* < .001)**
	No	3865 (83.1)	47 (22.1)		
Invasive mechanical ventilation	Yes	225 (4.8)	180 (84.5)	**107.32 (73.30**–**161.73, *p* < .001)**	**42.52 (27.39**–**67.93, *p* < .001)**
	No	4427 (95.2)	33 (15.5)	–	

NEWS: National Early Warning Score; CALL: Comorbidity − Age − Lymphocyte Count − Lactate dehydrogenase Score; WBC: white blood cell; MLR: monocyte to lymphocyte counts ratio; NLR: neutrophil to lymphocyte ratio; dNLR: derivate neutrophil to lymphocyte ratio; PLR: platelet to lymphocyte ratio; HFNC: high flow nasal cannula; IMV: invasive mechanical ventilation.

^a^Multivariate model 1: Number in model: 4793 / 4865; AIC = 1453.4, C–zstatistic = 0.818; H&L Chi-sq(8) 6.69 (*p* = .570).

^b^Multivariate model 2: Number in model: 4364 / 4865; AIC = 880.3, C-statistic = 0.956; H&L Chi-sq(8)25.32 (*p* = .001).

The values in boldface fonts are used for the correspondent significant (*p* < 0.05) probability tests.

**Figure 1.**
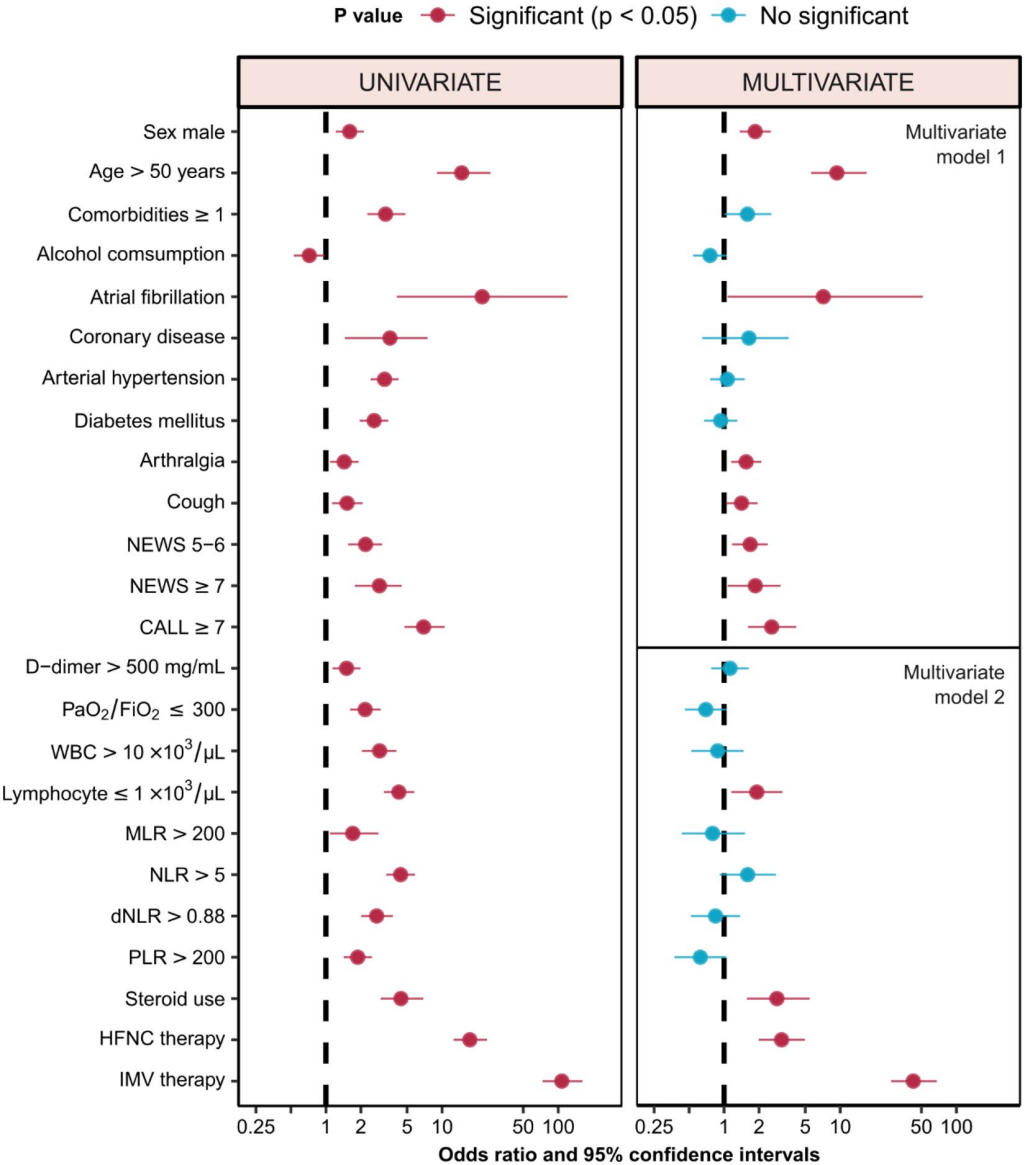
Odds ratio and 95% confidence intervals of basal, clinical, biochemical, and therapeutic characteristics with predictive value for mortality. NEWS: National Early Warning Score; CALL: Comorbidity − Age − Lymphocyte count − Lactate dehydrogenase Score; WBC: white blood cell; MLR: monocyte to lymphocyte counts ratio; NLR: neutrophil to lymphocyte ratio; dNLR: derivated neutrophil to lymphocyte ratio; PLR: platelet to lymphocyte ratio; HFNC: high flow nasal cannula; IMV: invasive mechanical ventilation.

In the univariate analysis, within the baseline characteristics, male sex (odds ratio [OR] 1.60; 95% CI: 1.21–2.13; *p* = .001), age ≥ 50 years (OR 14.75; 95% CI: 9.01–26.11; *p* < .001), have at least one comorbidity (OR 3.26; 95% CI: 2.27–4.83; *p* = .001), present atrial fibrillation (OR 22.14; 95% CI: 4.08–120.23; *p* < .001), and symptoms such as cough (OR 1.51; 95% CI: 1.13–2.07; *p* = .006) and arthralgias (OR 1.44; 95% CI: 1.09–1.91; *p* = .012) were independently associated with death. In addition, it was observed that the group of patients at high risk of progression with a CALL score ≥ 7 (OR 2.89; 95% CI: 1.78–4.50; *p* < .001), those who presented PaO_2_/FiO_2_ ratios ≤ 300 (OR 2.18; 95% CI: 1.62–2.96; *p* < .001), leukocyte count> 10 × 10^3^/μL (OR 2.90; 95% CI: 2.04–4.05; *p* < .001), lymphocyte count ≤ 1 × 10^3^/μL (OR 4.24; 95% CI: 3.16–5.77; *p* < .001), or NLR index > 5 (OR 4.40; 95% CI: 3.32–5.87; *p* < .001), they also had a higher risk of death. Within the treatment variables, having required steroid (OR 4.42; 95% CI: 2.96–6.90; *p* < .001), supplemental oxygen with high-flow nasal cannula (OR 17.35; 95% CI: 12.54–24.44; *p* < .001) or by invasive mechanical ventilation (OR 107.32; 95% CI, 73.30–161; *p* < .001), was also independently associated with an increased risk of death (Table 4, Figure 1). Null or low schooling at primary (elementary), compared with a higher degree of schooling, was independently associated with death (OR 1.93; 95% CI: 1.37–2.66; *p* = .0001); This association was also observed when comparing the group of patients with zero education against the rest with some degree of education (OR 3.47, 95% CI: 1.30, 7.73; *p* = .005).

In multivariate analysis, it was performed using two models, grouping baseline sociodemographic and clinical variables (model 1) and biochemical and therapeutic variables (model 2). In model 1, an increase in the risk of death was observed for men (OR 1.85; 95% CI: 1.36–2.53; *p* = .0001), age ≥ 50 years (OR 9.36; 95% CI: 5.60–16.83; *p* < .0001), suffer atrial fibrillation (OR 7.15; 95% CI: 1.07–51.41; *p* = .039), have a cough (OR 1.41; 95% CI: 1.04–1.95; *p* = .032) and arthralgias (OR 1.55; 95% CI: 1.15–2.09; *p* = .004) as symptoms, as well as scores ≥ 7 on the CALL scales (OR 2.57; 95% CI: 1.59–4.15; *p* < .001) or NEWS (OR 1.86; 95% CI: 1.07–3.07; *p* < .020). Having one or more comorbidities had a borderline value very close to statistical significance with OR 1.59 (1.00–2.55, *p* = .050).

In model 2, it was observed that, from the laboratory results, only the lymphocyte count ≤ 1 × 10^3^/μL remained as a significant risk factor with OR 1.91 (95% CI: 1.15–3.18; *p* = .012). Finally, in the multivariate analysis of the treatment variables, having required steroid (OR 2.85; 95% CI: 1.57–5.47; *p* = .001), supplemental oxygen with a high-flow nasal cannula (OR 3.12; 95% CI: 1.99–4.97; *p* < .001) or merited invasive mechanical ventilation (OR 42.52; 95% CI: 27.39–67.93; *p* < .001), was associated with a higher risk of death (Table 4, Figure 1).

## Discussion

Alternative care sites (ACS) are buildings that have been converted into medical care facilities for inpatients and outpatients to mitigate the impact of danger when patient volume exceeds available capacity [26]. Their optimal operation requires continuous preparation actions that are agile and adaptable, as well as an operating model that enables them to operate without jeopardizing safe and effective care, all while focussing on health equity, including the most vulnerable and marginalized populations, racial or ethnic minorities, and those without medical insurance [9]. These social determinants have been identified as critical factors in the occurrence, progression, and prognosis of SARS-CoV-2 infection, and thus warrant special attention [27–30].

4.37% of the COVID-19 patients treated in the UTC-19 died. According to the Health Secretariat, Mexico registered maximum mortality in January 2021, especially in the first three weeks of that month. This observation coincides with our results that show a local maximum percentage (7.14%) during this period, corresponding with the national ‘second wave’.

When studying the mortality between several ACS, we observe that the results are diverse even between studies in the same country. In Brazil [18], the Riocentro Campaign Hospital in Rio of Janeiro had a mortality of 25.5% (194/761 patients) and was associated with age and pulmonary impairment. In Poland [16], the National Stadium in Warsaw had a mortality of 8.91% (156/1749 patients), identifying 14 factors (some comorbidities, stroke history, chronic obstructive pulmonary disease, and heart failure) that have a significant impact on the prognosis and mortality. In South Africa [17], the International Convention Centre in Cape Town had a mortality of 5.7% (83/1502 patients), highlighting that the daily communication with the whole care service platform was a critical success factor. In China [13], the Leishenshan Hospital in Wuhan had a mortality of 2.3% (46/2011 patients), referring that the patients with intensive care unit (ICU) admission had more high mortality in contrast with the patients attended in a general ward (GW), with 41.8% in ICU versus 0.4% in GW. In the United States of America [19], the Javits Convention Center located in Manhattan, New York, had a mortality of 0.54% (6/1096 patients), with most patients receiving in the convalescent phase of their disease. In Spain, the IFEMA Exhibition Center in Madrid [14] had a mortality of 0.42% (16/3817 patients), and the H144 Hospital of the Health Service in Asturias [15] had a mortality of 17% (56/334 patients). The IFEMA Exhibition Center considered critical decisions in the management of patients with COVID-19, including several levels of care using clinical profiles based on comorbidities, oxygen saturation, respiratory rate, and evolution, and considered that the patients with a good baseline situation and high oxygen requirements, who might require admission to the critical care unit, should not be admitted to these centres [14].

It is essential to point out the limitations of carrying out a comparative analysis of mortality rates since the ACS around the world operated at several moments of the pandemic, between different periods, with a variety of admission and referral criteria, infrastructure, installed operative capacities, and particular care objectives for each region.

Additionally, the study of Jiménez et al. [31], reported differences between 30-day in-hospital unadjusted mortality in patients admitted in temporary and conventional ICUs (94/326, 28.83% for conventional vs 162/450, 36.0% for temporary, log-rank test *p* = .023, chi-squared *p* = .036) and that the hospitalization in temporary ICUs was an independent risk factor associated with mortality (hazard ratio, 1.4; CI, 1.06–1.83; *p* = .016). These authors indicated that a plausible explanation for the survival differences might lie in the allocation of specialized personnel among areas. The recommended nurse-to-patient ratio for an intubated patient ranges between 1:1 and 1:2, and presumably, the high workload of ICU nurses could have impacted events not assessed in that study.

In this study, hospital mortality was associated with sociodemographic variables such as sex (male), age (≥ 50 years), and low school level (zero to basic). Various meta-analyses have documented the association between mortality and the variables of sex and age [32,33], although with a lower cut-off point than that found in many of them (≥ 65 years). Younger age as a risk factor coincides with that reported by Biswas et al. [34], whose meta-analysis showed that men with COVID-19 had a significantly higher risk of mortality compared to women (relative risk [RR], 1.86; 95% CI: 1.67–2.07; *p* < .00001) and patients aged ≥ 50 years were associated with a significantly higher mortality risk 15.4 times compared to patients aged < 50 years. In the multivariate analysis of this study, we found a 9.36-fold increase in the risk of death for patients aged 50 years or older (95% CI: 5.60–16.83; *p* < .0001), positioning this condition as the baseline variable with the greatest weight to predict the outcome, above sex, comorbidities, symptoms, and the score of the prognostic scales.

Regarding the level of education, this study found an increase in the risk of death by 3.47 times for patients with zero education compared to the rest. Some studies have pointed out the disproportionate impact on mortality of people with less education [28], documenting that up to 25.6% of deaths occurred in individuals with an education level lower than high school (95% CI: 23.4–27.9%; *p* < .001). In Mexico, it was reported that in 2020 up to 71% of those killed by COVID-19 had a primary education or less (incomplete elementary school, preschool, or no education). These results allow us to glimpse the direct effects of the social determinants of health on vulnerability to infection. The factors involved include overcrowding, barriers to accessing health services, and financial difficulties that may force occupational exposure to continue [35,36].

In this study, having one or more comorbidities was associated with a 3.26-fold increase in the risk of death, however, in the multivariate analysis, it had a borderline value very close to statistical significance with OR 1.59 (1.00–2.55, *p* = .050). Atrial fibrillation, coronary artery disease, systemic arterial hypertension, and diabetes mellitus all had a significant association in the univariate analysis; however, in the multivariate analysis, only atrial fibrillation had a significant association. These results contrast with the international literature, especially in groups of older adult patients [37]. It has been postulated that these differences between studies may be due to the fact that the prognostic effect of clinical conditions on mortality from COVID-19 varies substantially according to the mean age of the patients [38].

In this study, it was relevant that there was no significant difference in the outcome for other comorbidities reported in the literature [39–42], such as active cancer (*p* = .07), liver disease (*p* = 1), chronic kidney disease (*p* = .07), chronic lung disease (*p* = .41), overweight (*p* = .57) and obesity (*p* = 1), although for many of these variables the representation of cases in which the disease occurred and death also occurred was null or very low.

In terms of symptoms, this study discovered that cough and arthralgia were independently associated with death, remaining significant after adjusting for sex, age, comorbidities, and clinical data at admission. This contrasts with the case series report on a protective factor for admission to the Intensive Care Unit [43] and death for patients with arthralgia (OR 0.31; 95% CI: 0.12–0.70). Both symptoms have biological plausibility; cough can be explained by pulmonary involvement and arthralgias by viral action and the release of pro-inflammatory cytokines [44,45]. Related to this, the meta-analysis published by Izcovich et al. [46], found that arthralgias/myalgias were a factor of risk for developing severe COVID-19 (OR 1.29; 95% CI: 1.03–1.61), however, it did not reach significance as a prognostic factor for mortality (OR 0.96; 95% CI: 0.77–1.23).

Establishing the degree of severity and prognosis of patients with COVID-19 is important to determine their site of care and treatment. In this study, the difference in CALL values (*p* < .0001, 95% CI: 2.48 to 3.09 points less in patients without death) and NEWS (*p* < .0001; 95% CI: 0.72 to 1.39 points less in patients without death). Although the authors of the CALL scale demonstrated the prognostic power of their instrument to predict progression to clinical worsening with an area under the receiver operating characteristic curve (AUROC) of 0.91 (95% CI: 0.86–0.94), some studies have indicated a low prediction of progression for the severity with the area under the curve (AUC) 0.62 (95% CI: 0.53–0.68), however, with high predictive power for hospital mortality with AUC 0.76 [46–49].

In this study, it was observed that the group of patients at high risk of progression with a CALL score ≥ 7 points had a higher risk of mortality with OR 2.89 (95% CI: 1.78–4.50; *p* < .001), remaining significant when adjusting for sociodemographic variables, comorbidities, and symptoms (OR 2.57; 95% CI: 1.59–4.15; *p* < .001).

Thus, while it has been demonstrated that age, lymphocytes, and LDH are reliable predictors of disease progression, the presence of comorbidity alone does not appear to be a reliable independent risk factor for disease progression [49], which is consistent with some predictive models that have also failed to establish comorbidity as a predictor of disease severity, which requires adequately validating the effect of previous diseases in patients [50,51].

Regarding laboratory results, this study documented biochemical variables that coincide with what was reported in a meta-analysis on their role as predictors of severity and mortality, including the PaO_2_/FiO_2_ ratio ≤ 300 [52,53], and the D-dimer > 500 mg/mL [54,55], increased leukocytes [56], and decreased platelet counts [57], and lymphocytes [58,59], lymphopenia being the one with the highest odds ratio for fatal outcome in this study (OR 4.24; 95% CI: 3.16–5.77; *p* < .001), and also the only parameter with statistical significance in the multivariate analysis, being noteworthy that the absolute lymphocyte count in the group with death was almost half of what was found in the group of survivors (*p* < .0001).

Neutrophil to lymphocyte ratio (NLR) is an inflammation biomarker, proposed in some meta-analyses to evaluate the dysregulation of the immune response linked to the development of viral hyper inflammation, predictive of severity and mortality in patients with COVID-19 [60,61]. In this study, the patients with NLR> 5 had a higher risk of death (OR 4.40; 95% CI: 3.32–5.87; *p* < .001). In the meta-analyses published in this regard, significant heterogeneity has been observed in the studies used, requiring statistical adjustments [62,63]; regardless of the different cut-off values of NLR, the relative risk of mortality pooled in patients with elevated levels of NLR versus normal tends to be significant (RR, 2.74; 95% CI: 0.98–7.66). In some studies, the index was calculated by inverting numerator and denominator (Lymphocyte to Neutrophil Ratio, LNR), observing that the ratio decreased significantly up to four times and, with an LNR cut-off value ≤ 0.088, was able to predict hospital mortality from COVID-19 with a sensitivity of 85.0% and a specificity of 74.2% [64,65]. Additional studies are required to determine the optimal cut-off value for NLR before of widespread clinical use.

Finally, this study analysed the treatment variables and discovered that, both in univariate analysis and in the multivariate model adjusted for biochemical parameters, requiring steroids, supplemental oxygen with high-flow nasal cannulas, or invasive mechanical ventilation was independently associated with an increased risk of death, with the latter increasing the risk of death by up to 425 times that of patients who did not require advanced airway management.

The main limitation of this study is that it was only one centre was included. Likewise, one of the main elements that have made it difficult to contrast with other studies is the fact that a high number of patients with low clinical risk were counted from the beginning due to the very nature of the ACS from which the data came. Even so, it has been seen that variables that generally have a prognostic value either to a fatal outcome or to disease progression, have had a similar characterization in this study.

## Conclusion

UTC-19 was one of the ACS with the greatest infrastructure and operating time in the world, enabling the response capacity to be expanded during the pandemic’s most critical phase in Mexico City. This study identified the clinical characteristics and risk factors for mortality associated with COVID-19, which enables the development of continuous preparedness actions and the improvement of the operational model while considering the social determinants of health emergencies.

## Supplementary Material

Supplemental Material

## Data Availability

The datasets used and analysed during the current study were collected as non-public data under the responsibility of the Temporal Healthcare Unit Administration at Mexico City. The data can be shared with the previous approval of the request.
