# Supplementary material for: Sociodemographic, clinical and laboratory characteristics and risk factors for mortality of hospitalized COVID-19 patients at alternate care site: a Latin American experience
Source: Ann Med. 2023 Jun 15;55(1):2224049. doi: 10.1080/07853890.2023.2224049 (PMC10281393; doi:10.1080/07853890.2023.2224049)
Supplement: Supplemental Material [file IANN_A_2224049_SM0468.pdf]

Additional file 1. Figure S1. Approval by the Research Ethics Committee. Research Division of the National Autonomous University of Mexico

UNIVERSIDAD NACIONAL AUTÓNOMA DE MÉXICO

DIVISIÓN DE INVESTIGACIÓN
FACULTAD DE MEDICINA
UNAM

COMITÉ DE ÉTICA DE LA INVESTIGACIÓN

OFICIO NO. **FMED/CEI/MHU/055/2020**

ASUNTO: **Dictamen Proyecto FM/DI/098/2020**

Dr. Rafael Ricardo Valdez Vázquez
Investigador Externo
Unidad Temporal Covid-19 Centro Citibanamex
P r e s e n t e.

Estimado doctor Valdez Vázquez:

Su proyecto **FM/DI/098/2020** de título **"Estudio de cohorte de pacientes infectados por SARS-CoV-2, atendidos en el "Centro COVID-19 Citibanamex" de la Ciudad de México: análisis comparativo de características sociodemográficas y clínicas en China, España y Brasil"** en su versión del 05 de octubre de 2020, ha sido **APROBADO** por el Comité de Ética de la Investigación, en sesión ordinaria de fecha **06 de octubre de 2020**, teniendo en consideración el dictamen aprobatorio de la Comisión de Investigación.

- La vigencia del proyecto será de **tres años** contado a partir de la fecha de la sesión.
- El proyecto deberá ejecutarse de acuerdo con lo especificado en la versión aprobada.
- Es necesario que envíe al correo de las Comisiones, un informe anual de avance en formato PDF. De lo contrario, se podrá cancelar la vigencia del proyecto.
- Deberá notificar cualquier modificación o enmienda al mismo y esperar la autorización correspondiente.

Sin otro particular, aprovecho la oportunidad para enviarle un cordial saludo.

A T E N T A M E N T E
"POR MI RAZA HABLARÁ EL ESPÍRITU"
Ciudad Universitaria, Cd. Mx., a 07 de octubre de 2020.

LA PRESIDENTA

Marcia Hiriart U.

DRA. MARCIA HIRIART URDANIVIA

*MHU/ETF.

UNAM La Universidad de la Nación

Additional file 2. Supplementary Table S1.

Optimal cut-points on selected numerical variables.

**Types:**	**Outcome: Negative - Cases without death; Positive - Cases with death**											
**Predictor**	**Age**	**NEWS**	**CALL**	**D-Dimer**	**PaO_2 / FiO_2 (%)**	**DHL**	**leukocytes**	**Linphocytes**	**1000 M:L ratio**	**N:L ratio**	**Derived N:L ratio**	**P:L ratio**
**Direction**	≥	≥	≥	≥	≤	≥	≥	≤	≥	≥	≥	≥
**Optimal cut-off point**	**54**	**4**	**8**	**320**	**289.2**	**198**	**8.1**	**1.0132**	**398.6711**	**3.5305**	**0.9252**	**226.3274**
**Area under the ROC Curve (AUC)**	**81.89%**	63.81%	**80.48%**	59.43%	62.08%	63.38%	60.43%	73.01%	65.51%	74.83%	67.31%	61.91%
**Method**	Maximize the metric function											
**Metrics**	Sum of the specificity and sensibility											
**Metrics Value**	1.4805	1.1841	1.4988	1.1406	1.2103	1.2017	1.1631	1.3586	1.2523	1.378	1.3096	1.1948
**Accuracy**	0.6232	0.6097	**0.7178**	0.4010	0.5882	0.5365	**0.7951**	0.6351	0.6063	0.5851	**0.7511**	0.6541
**Specificity (S_1) = TN / N**	0.612	0.6113	0.7147	0.3847	0.5862	0.5303	**0.8157**	0.6309	0.6044	0.5752	**0.7603**	0.6596
**Sensibility (S_2) = TP / P**	0.8685	0.5728	**0.784**	0.7559	0.6238	0.6714	0.3474	0.7277	0.6479	**0.8082**	0.5493	0.5352
**True negatives (TN)**	2847	2844	3325	1787	2447	2467	3788	2935	2807	2671	3531	3063
**False positive (FP)**	1805	1808	1327	2858	1725	2185	856	1717	1837	1973	1113	1581
**False negative (FN)**	28	91	46	52	76	70	139	58	75	42	96	99
**True positive (TP)**	185	122	167	161	126	143	74	155	138	171	117	114
**Youden's Index $= S_2 + S_1 - 1$**	**0.4805**	0.1841	**0.4987**	0.1406	0.2103	0.2017	0.1631	0.3586	0.2523	0.3834	0.3096	0.1948
$FI's\ Score = \frac{2\times TP}{2\times TP+FP+FN}$	0.1680	0.1139	**0.1957**	0.0996	0.1227	0.1126	0.1295	0.1487	0.1261	0.1451	0.1622	0.1195
**Gini's Index $= 2\times AUC - 1$**	**0.6378**	0.2762	**0.6096**	0.1886	0.2416	0.2676	0.2086	0.4602	0.3102	0.4966	0.3462	0.2382

Comments on the table of optimal cut-off points:

This table presents the summary of numerical variables considered in the study of odds ratios, to establish in more detail, the behavior of the variables used. It is emphasized that the cut-off points are like those handled in the said table; Although some values are very dissimilar, the adjustment has also considered their practicality, and the fact that there is contrast information in which cut-off points are already used is found. For this analysis, the cut point library (v. 1.1.1, Thiele and Hirschfeld, 2021) in R v.3.6.3 has been used, leaving the sum of sensitivity and specificity as a metric, although this function allows occupying several more metrics including some based on smoothing of the distributions by analyzed condition.

D-dimer concentrations have the lowest predictive value with an AUC = 59.43, considering that the test for this variable is poor to discriminate between patients with and without death, even though it could be used to discriminate positive cases in around 76% of the cases (sensibility = 0.7559).

Most of the variables analyzed present an AUC of 60% to 70%, considering that their use to discriminate between patients with and without death is regular, of these variables only the derived neutrophil-lymphocyte ratio (dRNL) presents a precision above 75% (precision = 0.7511), with negative discrimination above 76% (specificity = 0.7603), while the other variables in this group present a precision and sensitivity close to or below 65%.

The two numerical variables with the highest predictive value, according to the area under the curve presented, were the CALL score (AUC = 80.48%) and age (AUC = 81.89%), age presenting greater discrimination of positives (sensitivity = 0.8685) although with a precision of 62%, while the CALL scores presented discrimination of both positive cases (cases with death) and negative cases (cases without death) more or less adequate, with precision and sensitivity values above 0.71.

Reference:

Thiele, C. and Hirschfeld, G., 2021. cut point: Improved Estimation and Validation of Optimal Cut points in R, Journal of Statistical Software, 98(11), pp. 1–27. doi: 10.18637/jss.v098.i11.
